# Timing of head turns to upcoming talkers in triadic conversation: Evidence for prediction of turn ends and interruptions

**DOI:** 10.3389/fpsyg.2022.1061582

**Published:** 2022-12-20

**Authors:** Lauren V. Hadley, John F. Culling

**Affiliations:** ^1^Hearing Sciences – Scottish Section, School of Medicine, University of Nottingham, Glasgow, United Kingdom; ^2^School of Psychology, Cardiff University, Cardiff, United Kingdom

**Keywords:** conversation, prediction, head movement, communication, unaddressed listener

## Abstract

In conversation, people are able to listen to an utterance and respond within only a few hundred milliseconds. It takes substantially longer to prepare even a simple utterance, suggesting that interlocutors may make use of predictions about when the talker is about to end. But it is not only the upcoming talker that needs to anticipate the prior talker ending—listeners that are simply following the conversation could also benefit from predicting the turn end in order to shift attention appropriately with the turn switch. In this paper, we examined whether people predict upcoming turn ends when watching conversational turns switch between others by analysing natural conversations. These conversations were between triads of older adults in different levels and types of noise. The analysis focused on the observer during turn switches between the other two parties using head orientation (i.e. saccades from one talker to the next) to identify when their focus moved from one talker to the next. For non-overlapping utterances, observers started to turn to the upcoming talker before the prior talker had finished speaking in 17% of turn switches (going up to 26% when accounting for motor-planning time). For overlapping utterances, observers started to turn towards the interrupter before they interrupted in 18% of turn switches (going up to 33% when accounting for motor-planning time). The timing of head turns was more precise at lower than higher noise levels, and was not affected by noise type. These findings demonstrate that listeners in natural group conversation situations often exhibit head movements that anticipate the end of one conversational turn and the beginning of another. Furthermore, this work demonstrates the value of analysing head movement as a cue to social attention, which could be relevant for advancing communication technology such as hearing devices.

## Introduction

### Turn taking in conversation

One of the most impressive feats of human cognition is our ability to converse, spontaneously and easily, with familiar and unfamiliar interlocutors alike. In a conversation, individuals alternate between listening to speech: comprehending the intended message and monitoring for appropriate times to respond, and taking on the role of talker: producing a message while concurrently monitoring the listener’s understanding. The ability to coordinate utterances with others on the fly is a striking skill that even young children are able to grasp (Garvey and Berninger, 1981), in spite of the highly complex cognitive processes involved (Levinson and Torreira, 2015).

Of particular note in conversation is the apparent seamlessness of turn taking, with the average gap between one talker ending and another beginning being <250 ms in English (Stivers et al., 2009). Given that it takes at least 600 ms to prepare even a simple utterance (Indefrey and Levelt, 2004), this speed of response suggests that a listener does not simply wait for a talker to finish before preparing to respond, but that they predict the talker’s end and prepare ahead of time. Indeed, a range of evidence from speech listening studies suggest that listeners make and use predictions. One body of work has found that listeners predict the next word that a talker will say (Kuperberg and Jaeger, 2016), with neural responses to utterances that are lexically predictable vs. unpredictable diverging more than a second before the end of an utterance (Magyari et al., 2014). Another has found that listeners predict the time at which utterances will end, relying on both lexicosyntactic information (De Ruiter et al., 2006; Magyari and De Ruiter, 2012) and turn-end prosodic information (Bögels and Torreira, 2015, 2021). However, these studies typically focus only on turn-end estimation, and do not address how prediction works in a conversation, during which interlocutors simultaneously listen to their partner and plan their response. One study of predictability that did include the need to respond found that predictable utterances led to quicker, but not more accurate, responses (Corps et al., 2018), suggesting that while predictability impacts turn taking, it may be through allowing listeners to plan their response earlier rather than improving prediction itself. Yet real conversation includes a range of additional communicative signals from both the talker and the other listeners (Hadley et al., 2022), such as body movement and gaze, which could provide additional information as to when a turn will end.

Recently, there has been some criticism of the idea that short turn-taking gaps in conversation imply prediction, since in real conversation interlocutors may not always be directly responding to a prior turn, but instead continuing their point from several turns ago (Corps et al., 2022). In such cases, the interlocutor need not predict a partner’s speech content since they have more turn switches over which to prepare their response, and the timing of their response could be triggered by the interlocutor stopping (though even this could additionally involve use of turn-completion cues to avoid coming in while the prior talker is still speaking—see Torreira and Bögels, 2022). Hence, finding ways of measuring prediction in conversation that do not involve a focus on the gaps between turns, but rather that focus on the behaviour of the listening interlocutors, could be particularly valuable.

Importantly, listeners that are simply following the conversation could also benefit from predicting the turn switch (from either the prior talker’s cues or the upcoming talker’s cues), in order to shift attention in time to follow the conversational flow. This situation allows the listener to use cues from the current talker, signalling that they are about to end, as well as cues from the upcoming talker, that they are about to start. Hence this provides a range of additional information to typical studies of prediction during speech listening. In a group conversation, there are multiple possible targets when a floor change occurs, and a variety of cues indicate who is going to be the one to take the floor (from verbal signals, to gaze, to posture; Petukhova and Bunt, 2009; Weiß, 2018). By recognising that a switch in talker is about to occur, and accurately assessing cues to indicate the next speaker, the listener can orient their eyes and head to better see, hear and engage with the upcoming speaker (rather than reactively responding once the new talker has begun). Indeed, given the importance of lip reading to speech intelligibility in background noise (Macleod and Summerfield, 1987), prompt reorientation of attention to a new speaker is probably vital to continued comprehension of a conversation in a noisy environment. An advantage of analysing the behaviour of third parties is that head and eye movements between the two speakers are not constrained by the need to avoid interruption; etiquette does not require the third party to wait until the current speaker has finished, so the behaviour can directly reflect the anticipated moment of the exchange of floor.

### Turn following in conversation

While orienting to the upcoming talker proactively may benefit the listener as discussed above, most studies have shown listeners to predominantly shift their gaze only after the first talker has finished their turn. A number of studies have investigated this issue using a videoed conversation protocol (Foulsham et al., 2010; Foulsham and Sanderson, 2013; Hirvenkari et al., 2013; Keitel et al., 2013; Casillas and Frank, 2017; Hidalgo et al., 2022), in which a group of conversing interlocutors are filmed, and the video is then presented to experiment participants while their gaze is tracked. When watching either dyads (Hirvenkari et al., 2013; Keitel et al., 2013; Casillas and Frank, 2017; Hidalgo et al., 2022), or larger groups (Foulsham et al., 2010; Foulsham and Sanderson, 2013), the timing of adult observers’ looks to the upcoming talker has most commonly been reported to occur during the gap between the offset of talker one and the onset of talker two (Foulsham et al., 2010; Keitel et al., 2013; Casillas and Frank, 2017; Hidalgo et al., 2022), or even after talker two has already begun (Foulsham and Sanderson, 2013; Hirvenkari et al., 2013). As most of these studies investigated younger adult participants (with only Hidalgo and colleagues also including older adults), differences between studies could relate to the duration of the gaps: switches are more likely to occur in the gap when longer gaps are used (e.g. in studies with longer gaps such as Keitel et al., 2013: or Hidalgo et al., 2022); or to the content/quality of the turns: question-answer pairings lead to earlier switches than other types of turn (Casillas and Frank, 2017), and more degraded audio leads to a delay (Hidalgo et al., 2022). Furthermore, it is important to note that many studies do not appropriately account for chance in terms of when gaze switches occur: only two studies calculated a chance-level baseline against which to compare the distribution of gaze switches (Hirvenkari et al., 2013; Casillas and Frank, 2017). In sum, videoed-conversation studies have not shown people to consistently orient to an upcoming talker before the prior talker is finished, though there is some evidence that they may nonetheless orient to the upcoming talker ahead of that talker’s utterance. But while the videoed conversation protocol benefits from the high level of constraint that can be applied to the conversation stimuli, it does designate the participant as a passive observer rather than an active interlocutor, potentially reducing their need (or motivation) to use conversational turn switch cues predictively.

Only one prior study (Holler and Kendrick, 2015) has used a real conversation protocol to investigate orientation switches, and involved adults ranging from 19 to 68 years old. In this paradigm, a group of participants converse, and the one inactive participant’s behaviour at each relevant turn switch is analysed. While this paradigm is of course much less controlled, it is more naturalistic, and engages all participants as interlocutors in the conversation situation. In this paradigm, the ‘observer’ may receive quite different (and enhanced) cues to the turn switch, as the group of interlocutors know that they are being observed and may provide additional cues to who has/is taking the floor to avoid overlap and conversation breakdown. By investigating gaze behaviour in seven triads, specifically during question-answer pairings, Holler and Kendrick found the modal timing of orientation to the upcoming talker to be 50 ms before the end of the prior talker’s turn, with over 60% of looks being predictive. When overlapping turns were removed, head turns on average occurred slightly later, peaking at 105 ms after the end of the prior talker’s turn. However, given that eye movements typically take 200 ms to prepare (Salthouse and Ellis, 1980), both of these findings were taken as evidence that interlocutors predict the end of a talker’s turn and pre-emptively shift attention to an upcoming talker.

The work of Holler and Kendrick (2015) therefore revealed a substantial number of looks to upcoming answerers to occur before the end of a talker’s question. However, several elements of their design may have encouraged predictive looks. Question-answer turn pairs have previously been demonstrated to be more strongly anticipated than other types of turn switches (see Casillas and Frank, 2017), as they may provide specific prosodic and syntactic cues to turn ends and the requirement to respond (Karttunen, 1977; Stivers and Rossano, 2010; Tomasello et al., 2022). Additionally, interlocutors were familiar, and such familiarity may have led to increased common ground (Fussell and Krauss, 1989), alignment or mimicry (Lakin and Chartrand, 2003), and thus prediction of their actions. Next, participants were conversing in ideal conditions: a quiet soundproof room. Work by Hidalgo et al. (2022) indicates that predictive looks decrease in degraded conditions, with the latency of the gaze switch increasing and relating to comprehension of the speech. Finally, gaze shifts were not assessed in relation to chance, and hence reported proportions may provide an overestimation of predictive looks to the upcoming talker. We addressed these possibilities by (1) analysing turns to upcoming talkers during all types of turn switches since question-answer pairs may make up only a minor proportion of turn switches (Forsyth and Martell, 2007), (2) analysing a dataset including only unfamiliar participants, (3) analysing the effect of noise level, and (4) including a measure of chance when assessing proportion of switches to the upcoming talker.

### Gaze vs. head turn in conversation

Both the head and the eyes can indicate an individual’s locus of social attention (Langton et al., 2000; Nummenmaa and Calder, 2009), and while prior research has focused on gaze to identify which talker somebody is attending to, in this paper, we focus on head orientation. Both the head and the eyes have been found to contribute to social attention, reflexively triggering shifts of an observer’s attention (Langton and Bruce, 1999; Pejsa et al., 2015), but there are a number of practical reasons to prefer use of head orientation to identify an interlocutor’s direction of attention. While the head and the eyes typically orient in the same direction during conversation (~70% of the time, Stiefelhagen and Zhu, 2002), the eyes also have other social functions, such as signalling or influencing dominance, competence, intimacy and liking (Kleinke, 1986; Kuzmanovic et al., 2009; Hamilton, 2016). Thus, the orientation of the head may be a more consistent signal to social attention, and recordings of head movement are less prone to dropout (since gaze is lost every time the participant blinks). Finally, the orientation of a listener’s head is already used to identify attention in hearing technology, with hearing aids in conversation situations prioritising sound coming from the front of the listener, demonstrating the practical importance of understanding head orientation behaviours.

### Predictive orientations

During a conversational turn switch in which there is no overlap between talkers, the listener’s orientation from one talker to another can occur at one of three points: (1) before the prior turn ends, (2) during the gap between the prior and upcoming turns or (3) after the upcoming turn has begun. Turns at different times may be driven by different processes. The simplest situation is (1) when the head turn occurs before the prior spoken turn ends. This provides clear evidence of the listener predicting the end of the talker’s turn and an imminent change of floor. The case of (2) is more complicated, and may or may not be considered evidence of prediction. If a listener turns their head during the time between the prior talker and the upcoming talker, i.e. during the gap, it could either indicate a prediction of the upcoming talker starting their turn: i.e. the listener using cues given by the person about to take the floor that signal they are about to begin, or it could be a simple reaction to the end of the prior talker’s turn: i.e. the listener using turn end cues to recognise that a talker is done. Yet even in this reactive case, turning towards the ‘correct’ upcoming talker may nonetheless still be considered to demonstrate the use of predictive cues to correctly identify who is going to start next (although this is less the case in a triad as there is only one possible responder aside from the participant themselves). Finally, situation (3) does not provide evidence for prediction, as it could be explained as a reactive response to the latter talker’s speech.

When there is an overlap between the speech of the prior and the upcoming talker, the behaviour indicating prediction would differ from the non-overlapping case discussed above. In this case, a second talker ‘interrupts’, and prediction would be demonstrated specifically by head turns towards the interrupter prior to them starting to talk. Such head turns would suggest that the listener is using cues given by the interrupter that signal they are about to begin, even though the prior talker has not finished. Any head turns occurring after the interrupter had begun could be a simple reaction to the new talker speaking.

### Motivation for the current study

Given prior evidence of prediction of speech, and the speed of conversational turn taking, interlocutors listening to a turn exchange may predictively orient to an upcoming talker to facilitate processing of their speech and thus more efficiently follow the conversation. In this study, we therefore investigate when unaddressed interlocutors shift their attention from one talker to another during a conversational turn switch. Since most prior work has focused exclusively on non-overlapping utterances and reported gaze switches typically occurring during the gap, with the only study demonstrating predictive gaze switches focusing exclusively on the more predictive question-answer pairings, there is currently no clear evidence regarding the use of prediction across general conversational turn switches. We specifically analyse the unaddressed participant across a large number of triadic conversation turn switches to assess evidence for whether and how often unaddressed interlocutors predict the end of the prior talker’s turn (in the case of non-overlapping speech), or the start of an interrupter’s turn (in the case of overlapping speech).

Furthermore, we analyse how background noise level affects predictive looks. Based on the recent work of Hidalgo et al. (2022), noise may reduce predictive orientations, with poorer or more effortful comprehension reducing the resources available for generating the predictions in the first place (Kuperberg and Jaeger, 2016). However, that study retrospectively degraded conversations that were recorded in quiet, yet it is possible that interlocutors holding real conversations in adverse conditions change their behaviours to emphasise turn-switch cues. If this is the case, observers may be able to make greater use of such cues, and may prioritise predictively turning to upcoming talkers in order to put themselves in the best position to process upcoming speech. We therefore compare predictive orientations across a range of noise levels reflective of conditions in which conversations are held in everyday life (Lebo et al., 1994).

As there are some inherent differences when watching a turn switch in a videoed conversation, and watching a turn switch between members of one’s own conversational group, we re-analyse the data of Hadley et al. (2021), in which previously unfamiliar participants took part in relatively free conversations in groups of three (allowing one participant to ‘observe’ each turn switch). During conversations, noise level varied between 54 and 78 dB, therefore ranging from relatively quiet to relatively loud. We also explore the effect on the analysis of assuming 200 ms of motor-planning time for a reorientation from one talker to another, as is common in studies of gaze switching (Salthouse and Ellis, 1980). We present data both in relation to turn end/start, as well as data in relation to the turn end/start plus 200 ms, to account for the fact that a movement would still have been ‘early’ with up to a 200 ms delay, when accounting for motor-planning time.

## Materials and methods

### Dataset details

Hadley et al. (2021) recorded head movement and speech data from free conversation in noise among 11 groups of three older adult participants arranged in an equilateral triangle (*N*_triads_ = 11, *M*_age_ = 61, SD_age_ = 11). Triads each engaged in four conversations lasting 12–16 min each: two in speech-shaped noise, and two in multi-talker babble noise. Noise level varied every 15–30 s between 54, 60, 66, 72, and 78 dB, as the original study was already designed to address coping strategies when communication varied from easy to challenging. Participants were unfamiliar. Data were collected from all three participants concurrently using a level threshold to automatically record the voice activity and an infra-red motion tracking system to record the head movement. In total, this dataset included 43 conversation recordings (one recording was lost due to technical difficulties); 22 in babble and 21 in speech-shaped noise with triads doing two sessions in each type of noise. With all three participants analysed of each triad, there are over 29 h of data in all.

### Identification of turn taking and related head movement

We extracted head movements consistent with turn-switch following from both datasets. An automatic algorithm developed in MATLAB applied the following processing stages. Note that separate analyses were run on non-overlapping speech (in which the turn of the prior talker was followed by the turn of the upcoming talker either immediately or following a gap) and overlapping speech (in which the turn of the prior talker is still ongoing when the upcoming talker begins).

#### Voice-activity detection

The voice-activity detection (VAD) data were based on sampled data from a close microphone. The data were pre-processed so that relatively clean exchanges of the conversational floor could be detected. The pre-processing was done in two stages. First, uninterrupted pauses in voice activity from a particular talker were closed up so that they were recorded as talking continuously. Second, any remaining voice activity whose duration was <0.5 s was deleted as representing only momentary isolated vocalisation (i.e. backchannels such a ‘mmm hmm’, or ‘right’) or perhaps mechanical noise on the microphone rather than full possession of the conversational floor. Speech onset and offset detection was then taken from the start and end points of each period of VAD.

#### Saccade detection

Head movement (HM) saccades were detected as movements of the head that exceeded 10° within a 400-ms window (i.e. a rate of movement averaging at least 25°/s over at least 10°). Having detected the presence of a saccade, the initiation and termination were defined as the points in time where the rate of movement fell to <5°/s over a 40-ms window.

Pairings between saccades between saccades and exchanges of conversational floor were also identified automatically. A ± 4-s interval preceding and following a saccade initiation was scanned for the nearest VAD onsets and offsets by the other two participants that were consistent with the direction of head movement.

**Figure 1:**
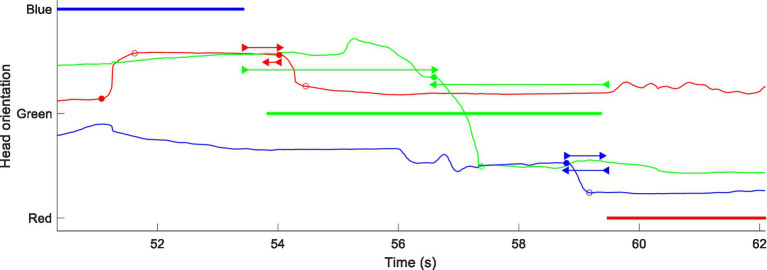
Example of Hadley et al. data (group #1 session #2 in babble, analysis of non-overlapping dialogue). Colour-coded VAD data (thick lines) with superimposed head-yaw data (thin lines) using the same colours of a given interlocutor. Head yaw has been transformed so that the curve moves coherently between the three VAD data lines for the respective talker directions. Detected head saccades are marked at their initiation (filled circles) and termination (open circles). Associations between saccade initiation and VAD offsets and onsets are marked by forward- and backward-pointing double-headed arrows, respectively.

The algorithm and its parameters were assessed for reliability by visual inspection of an automatically annotated visualisation. An example of such a visualisation is shown in Figure 1. The three participants, colour-coded as Red, Green and Blue, and their locations have been mapped onto the vertical axis such that the HM lines will shift to and from the VAD lines of the speakers towards whom they have oriented. Detected saccades and their associations with exchanges of the conversational floor have been marked.

In Figure 1, Red makes a movement *in response* to the exchange of floor between Blue and Green, starting at 53.7 s. Blue makes a movement *in anticipation* of an exchange from Green to Red at 58.7 s. The algorithm also detects Green moving between Blue and Red at 56.6 s. It is less clear that this is a true exchange of floor, because Green speaks during the gap between the other two voices. The algorithm accepts it, but the time intervals between the saccade and the associated offset and onset are both around 3 s, a time interval that tended to be classified as “coincidental” in the statistical analysis (see below).

### Statistical analysis

**Figure 2:**
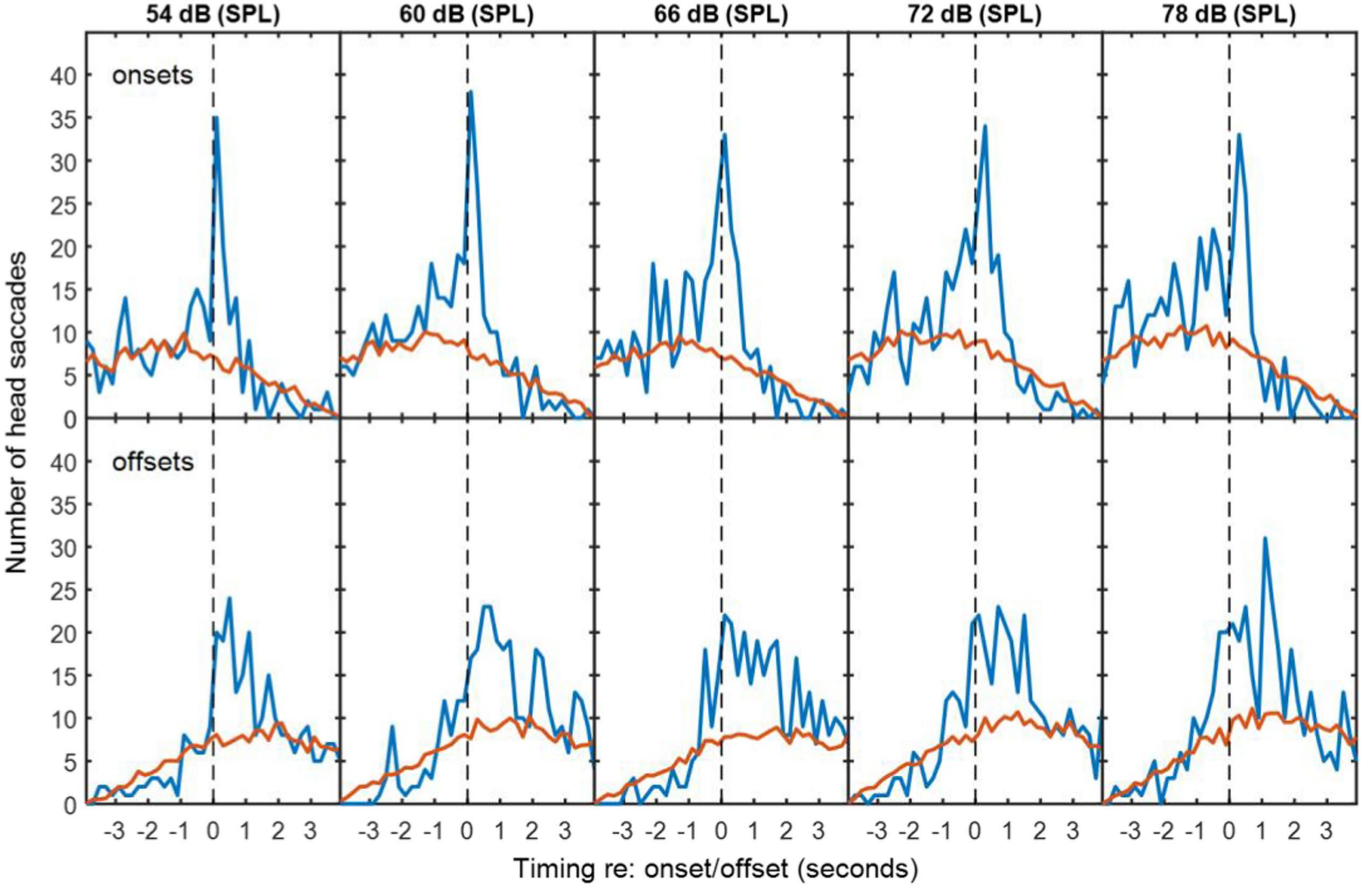
The top row of panels are histograms of number of saccades made by the participants in speech-shaped noise as a function of the time differences between these saccades and corresponding VAD onsets for the other two participants. Blue lines are for matched VAD and HM data. Red lines are for mismatched VAD and HM data, scaled to compensate for the number of different possible mismatches. Successive panels are for speech-shaped background noise at increasing sound levels. The bottom rows are similar histograms for the VAD offsets. The dashed vertical lines divide responses that preceded the VAD offset or onset from those that followed it.

**Figure 3:**
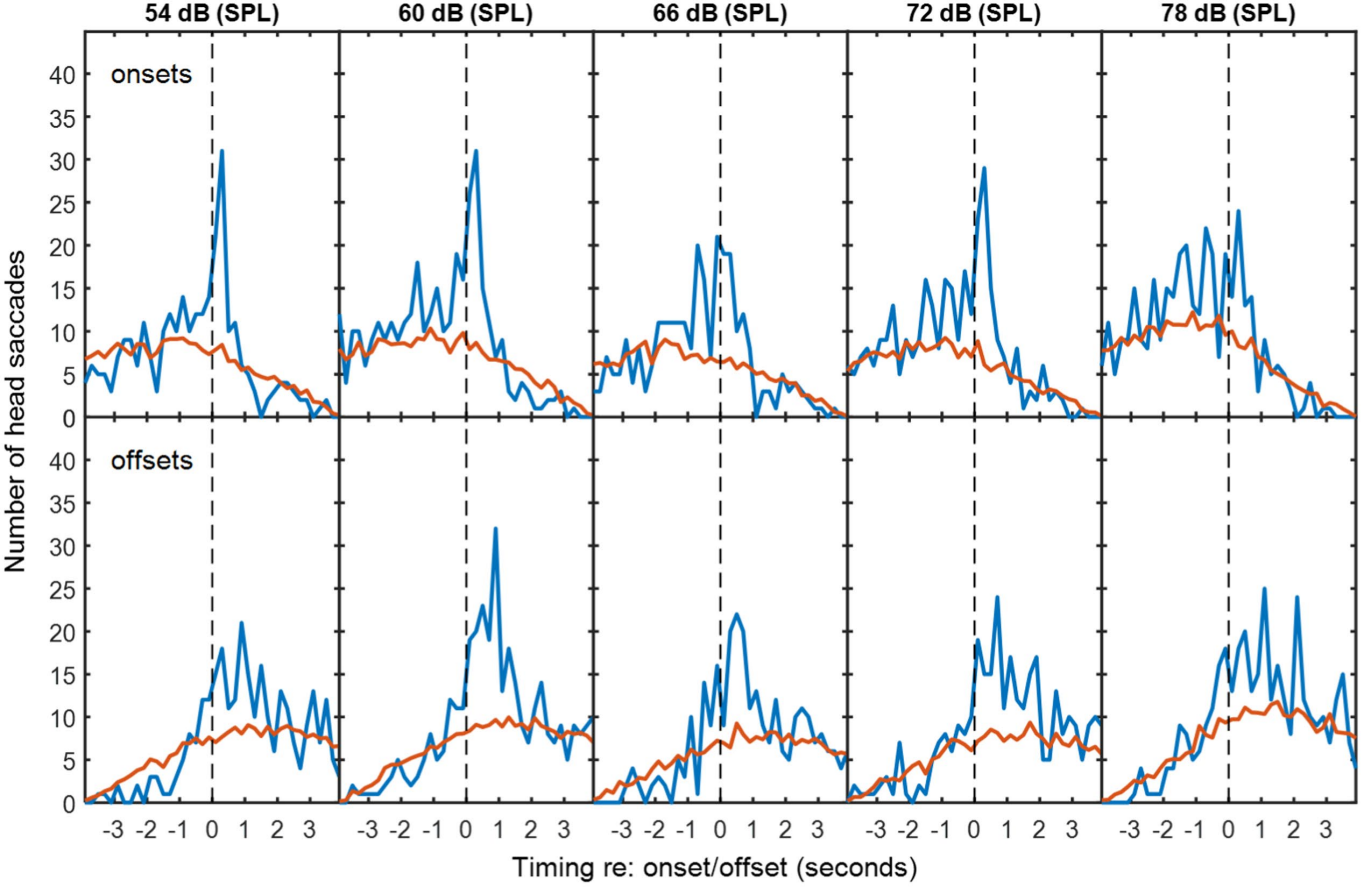
As Figure 2, but with multi-talker babble as background noise.

**Figure 4:**
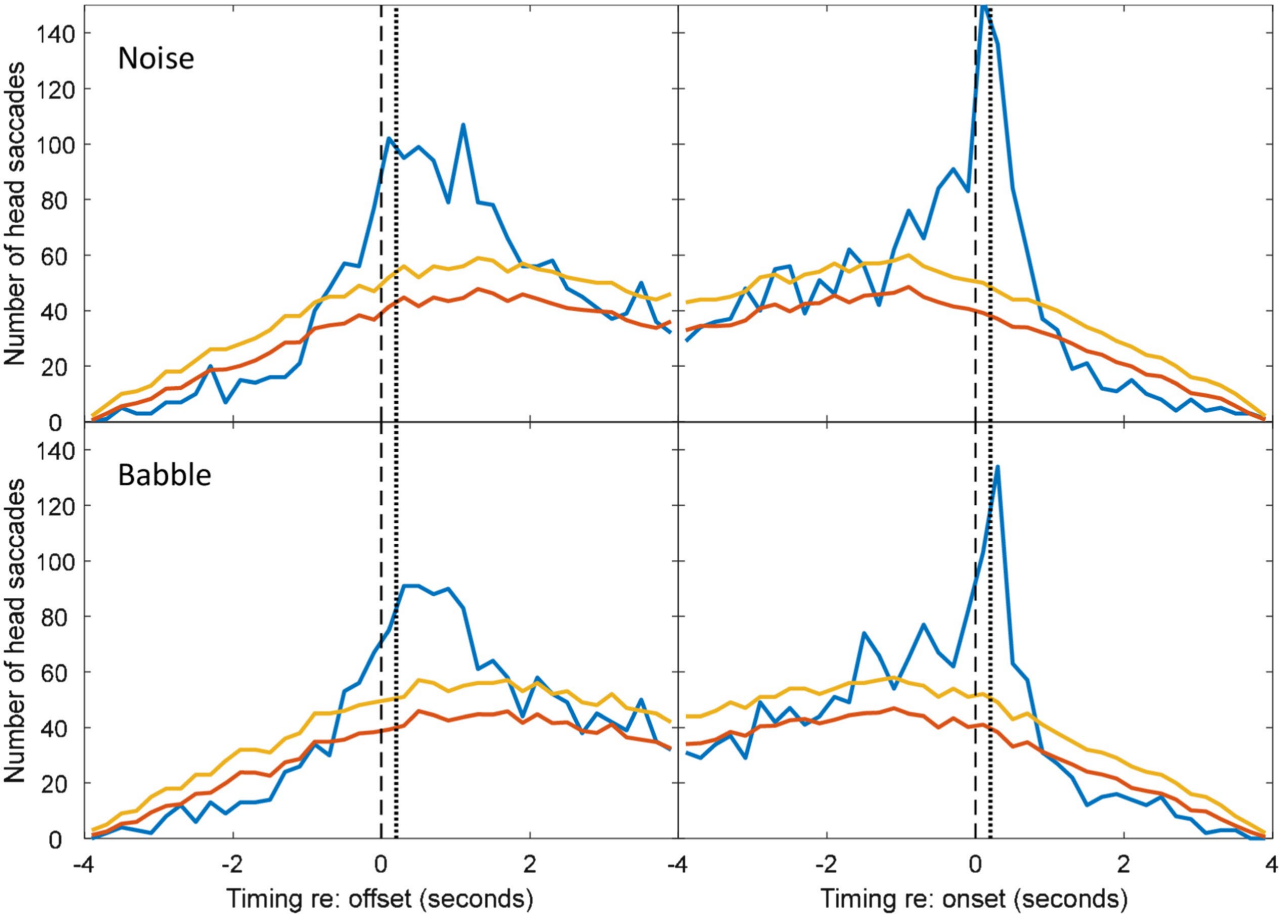
Histograms during non-overlapping utterances from Figures 2, 3.

To determine whether HMs were more frequent in time bins around the exchanges of floor than would occur by chance, the numbers of detected HMs were compared with mismatched VAD and HM data. Mismatched VAD and HM data were produced by pairing VAD and HM data from different sessions using all possible combinations with the same background noise type. The red lines in Figures 2–4 are thus the average numbers of head saccades paired with an exchange of conversational floor by chance, when the data are mismatched. The time bins are 200-ms in duration. The blue lines are the corresponding numbers of head saccades when the data are from the same session, so the difference between the two lines is the increase in head saccades caused by the corresponding exchanges of conversational floor, which we will term “associated” as opposed to “coincidental” HMs.

To determine whether the excess of associated HMs was statistically significant within each time bin, the null hypothesis was adopted that HMs are randomly distributed in time, with equal probability at a given time unit. However, the probability of a coincidental HM varies with respect to the timing of VAD onsets and offsets (see Figure 4) due to the saccade detection algorithm described above. The tail probability of a given number of head saccades in each time bin was calculated from binomial probability using the coincidental-HM probability for each time bin. The yellow lines in Figure 4 are the resulting critical number of head saccades for *p* < 0.05 (one-tailed) in each time bin.

The number of associated HMs in each time bin was estimated by subtracting the expected number of coincidental HMs (red lines) from the observed number of HMs (blue lines). These numbers were used to calculate the distribution in time of associated HMs. Negative values were discarded, because these may reflect a genuine suppression of movements prior to or following an onset or offset. In the figures presented below, data from all participants are pooled. To conduct the inferential statistics, data from each triad were pooled, because data from one individual were too sparse to generate a histogram. Mean and standard deviations of the associated saccade timings with respect to a VAD onset or offset were reconstructed from the difference between the expected coincidental HMs and the observed numbers for each triad in each condition. Two four-way repeated-measures ANOVAs were conducted: one addressed the mean timing of the HMs and one addressed the standard deviation of their timing. The first was intended to detect whether any factor influenced how promptly an HM was made, while the second was intended to detect whether any factor made listeners less precise in the timing of their movements. Both ANOVAs examined the factors of noise level, noise type (babble vs. speech-shaped noise), type of exchange (overlapping/non-overlapping) and event type (voice onset or voice offset). Follow-up statistics include simple main effects and Holm-corrected *t*-tests.

## Results

### Non-overlapping speech

In exchanges with non-overlapping speech, the median duration of silence between talkers was 0.64 s for speech-shaped noise and 0.68 s for babble. Figures 2, 3 show histograms of the timing of head movements with respect to offset and then onset of speech around these silences. The data are disaggregated by background noise level, for the speech-shaped noise (Figure 2, Table 1) and babble (Figure 3, Table 1). Elevation of the blue lines above the red indicates that the saccades in question are more frequent than might occur by chance and reflect, therefore, real reactions to exchanges of floor—associated HMs. The total estimate of associated HMs across the entire dataset exceeded 2,000. There tends to be evidence of associated HMs within about 2 s of an onset or offset. Outside this range, HMs are sometimes slightly below the expected chance level, particularly following an offset or preceding an onset.

It is noticeable that the red curves for the coincidental rate of HM detection are not flat, but peak near to zero lag and are also skewed differently for the onsets and offsets. These effects are to be expected from the HM-detection algorithm. The distribution has a peak near zero delay, because only the HMs closest to zero delay are associated with a given onset or offset; if there is a second-nearest HM, it will be ignored. The different skews for association with onsets and offsets are caused by the rejection of overlapping dialogue; because the 4-s analysis window is centred on the saccade under examination and the onset must always follow offset, the eligible onsets are necessarily later in time that eligible offsets.

The associated HM lags are predominantly positive for VAD offsets, suggesting that they are largely reactive or timed to occur at the moment a talker finishes saying something. The saccade lags are more evenly and precisely distributed around zero delay with respect to the VAD onsets, suggesting that they may be timed to coincide with the onset. The ANOVA of standard deviation confirmed an interaction between type of exchange and event type [*F*(1,10) = 10.2, *p* = 0.01], wherein the standard deviation is smaller (the timing is more precise) for onsets than offsets in non-overlapping dialogue (*t*(10) = 3.25, *p* < 0.05), but not in overlapping dialogue.

There is no obvious difference in the pattern of results as a function of noise level or noise type, but the ANOVA for the standard deviation of the HM timing (covering both overlapping and non-overlapping speech) revealed a small effect of noise level in which the timing was most precisely timed (i.e. a smaller standard deviation) at 60 dB (SPL) and became progressively less precise at higher noise levels [*F*(4,40) = 2.97, *p* < 0.05], reaching 10% larger at 78 dB. The only effect involving noise type was a four-way interaction on the mean timing of HMs [*F*(4,40) = 3.27, *p* < 0.05], but the nature of this interaction is unclear.

The five noise levels were then pooled in order to facilitate robust statistical analysis of anticipation. Figure 4 shows the pooled data for speech-shaped noise and babble, respectively. In the pooled data, the distributions of associated HMs around offsets and onsets are clearer. In speech-shaped noise, we found that 18% of associated HMs around an offset were made in advance, while the corresponding figure for babble was 20%. Assuming a motor-planning time of 200 ms, the number of HMs planned in advance of offsets increases to 28% and 23%, respectively.

Blue and Red lines are for matched and mismatched VAD and HM data. Yellow lines are the threshold of statistical significance (*p* < 0.05). The dashed vertical lines divide HMs that preceded the VAD offset or onset from those that followed it. The dotted vertical line is shifted by 200 ms in order to divide HMs that were planned before the VAD offset or onset from those that were planned after it. Elevations of the blue line above the yellow indicate a number of saccades that is significantly above chance (Figures 4, 7).

**Table 1:** Mean and standard deviations for associated HM delays (seconds) relative to nearest speech onsets and offsets for non-overlapping speech.

Noise level (dB)	Offsets				Onsets			
	Noise		Babble		Noise		Babble	
	Mean (s)	SD (s)	Mean (s)	SD (s)	Mean (s)	SD (s)	Mean (s)	SD (s)
54	0.61	1.07	1.07	1.10	−0.18	1.31	−0.12	0.91
60	1.00	1.3	0.81	1.02	−0.24	0.96	−0.54	1.28
66	1.04	1.13	0.58	1.06	−0.47	1.13	−0.30	0.91
72	0.68	1.13	0.98	1.48	−0.31	1.05	−0.33	1.13
78	0.68	1.12	0.92	1.36	−0.74	1.37	−0.72	1.22
Mean	0.81	1.16	0.87	1.21	−0.39	1.16	−0.40	1.09

### Overlapping speech

When the new speaker begins before the first speaker has finished, it is impossible to know whether a HM that occurs after this interruption is made in response to the onset or in anticipation of the offset. However, HMs made before either event might be anticipating an interruption. In a real 3-way conversation, there may be many linguistic and visual cues that may lead the observing party to make such a prediction. It is still, of course, possible that these responses are making a longer-range anticipation of the offset of the first speaker.

**Figure 5:**
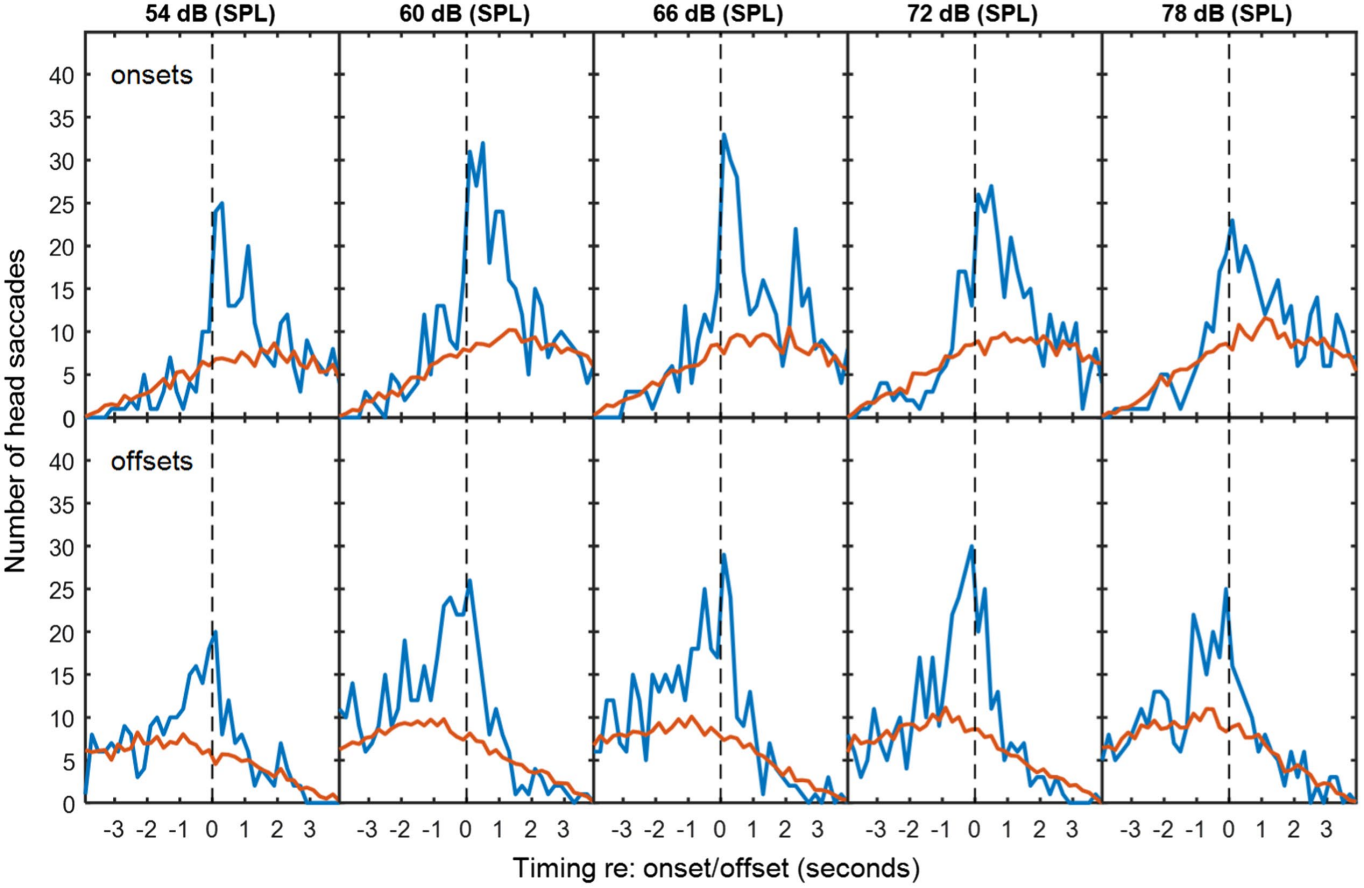
As Figure 2, but for overlapping dialogue in babble noise.

**Figure 6:**
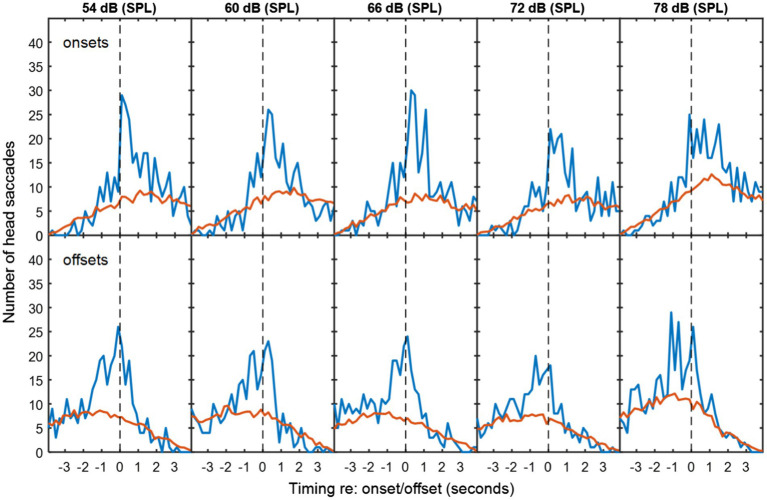
As Figure 3, but for overlapping dialogue in babble noise.

**Figure 7:**
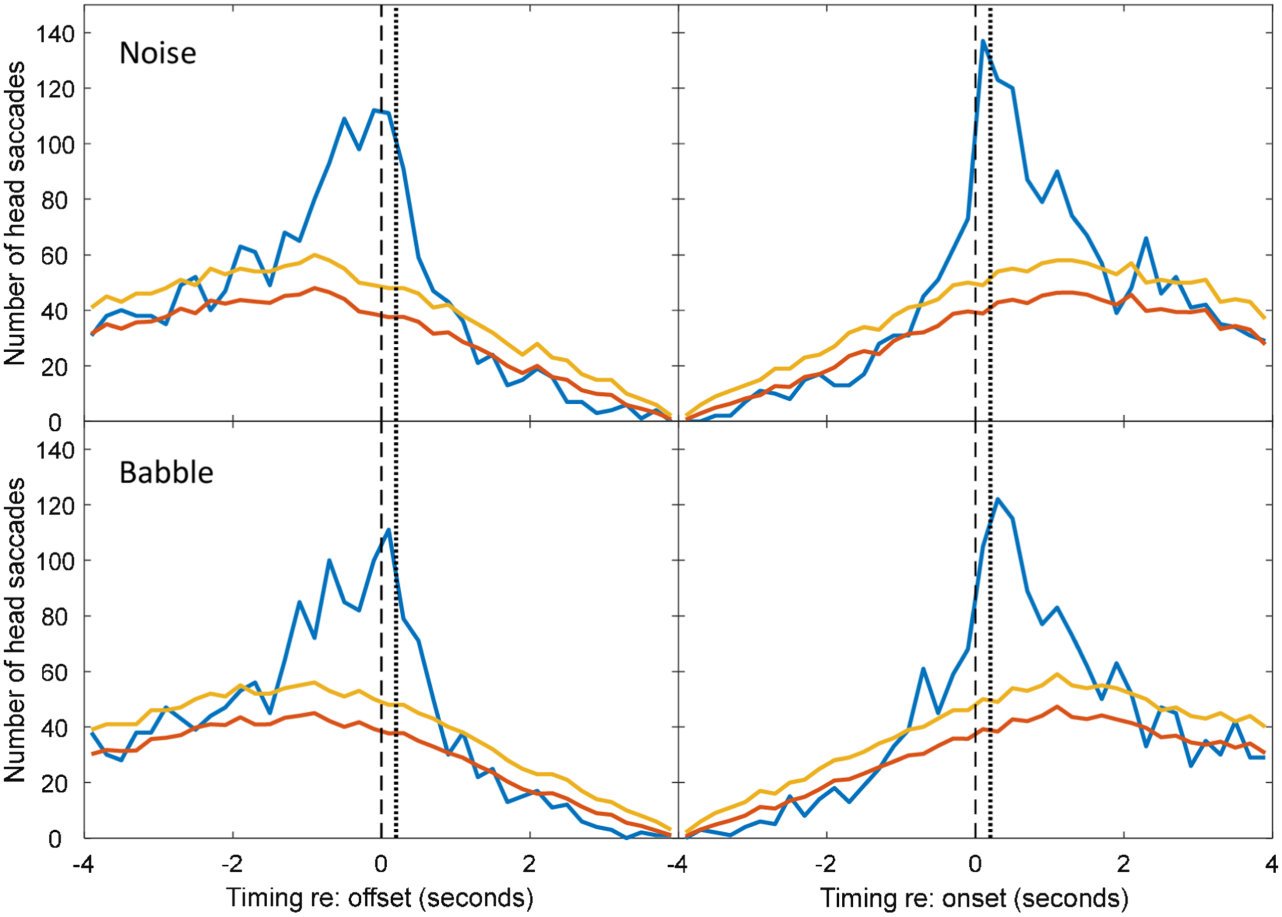
As Figure 4, but for head turns during overlapping utterances.

Figures 5–7 show the distribution of responses with respect to onsets and offsets for cases of overlapping data, pooled across noise levels. Using a similar analysis of associated HMs, we found that 16% of associated HMs preceded the onset of the new speaker in a speech-shaped noise and 20% in a babble noise. These numbers increase to 32% and 33% respectively, if it is assumed that listeners required 200 ms of motor-planning time to initiate the HM.

Compared to the data for non-overlapping dialogue in Figures 2–4, the pattern of temporal skew is reversed for the overlapping dialogue in Figures 5–7. HM times now tend to follow offsets and precede onsets. This effect may be expected from the fact that the onsets and offsets have themselves have reversed order between the two types of exchange. The ANOVA of mean HM time confirmed a significant interaction between the type of exchange and event type [*F*(1,10) = 1,837, *p* < 0.001]. In the interaction, simple main effects confirmed that head turns occurred earlier in relation to speech onset than offset for non-overlapping speech (onset = −584 ms, offset = 904 ms; *F*(1) = 362, *p* < 0.001), and earlier in relation to offset than onset for overlapping speech (offset = −696 ms, onset = 740 ms; *F*(1) = 488, *p* < 0.001). No other effects were statistically significant.

## Discussion

By analysing real triadic conversations between unfamiliar older adult participants, we demonstrated that a substantial proportion of head saccades from the unaddressed interlocutor around a turn switch occurred predictively. When conversational turns did not overlap, an average of 17% of the listener’s head turns to the upcoming talker occurred before the prior spoken turn ended, increasing to 26% when motor-planning time was taken into account. When conversational turns did overlap, an average of 18% of the listener’s head turns to the interrupter occurred before the interrupter interrupted, increasing to 33% when motor-planning time was taken into account. This is strong evidence for prediction of the turn end and the turn start, respectively.

It is important to acknowledge that whereas most work using videoed conversation shows reactive orientations to an upcoming talker (either occurring during the gap or after the upcoming talker has started), we and Holler and Kendrick (2015) showed a substantial proportion of predictive orientations. There are a few possible explanations for these differences between paradigms. First, in several of the studies using videoed conversations, the durations of the gaps were surprisingly long (Keitel et al., 2013: average gap 860–930 ms; and Hidalgo et al., 2022: average gap 500 ms), potentially reducing participants’ use of predictive turns since there is no need to orient quickly (and quickly turning from the current talker would in fact lead the participant to longer instances of boring/unanimated visual input). This is very different in a real conversation, when rapidly reorienting could support the listener to keep up and also convey a social function (of demonstrating attention). Second, when dyads are filmed and are only talking to each other (as in many of the videoed-conversation studies), there is less need for a listener to signal that they want to take the floor since they have no other interlocutors to compete with. In a real group conversation on the other hand (i.e. triads or larger, in order to have an unaddressed interlocutor), it is critical for a listener to signal when they want to claim the floor ahead of time since they have at least one other listener to compete with. A further possibility is that the talker in a larger group emphasises their cues to turn ends since they are communicating to a range of individuals, consistent with the work of Fay et al. (2000), who demonstrated that listeners show greater understanding for speech produced by talkers speaking in larger than smaller groups. Finally, we note that we specifically focused on older adults (and indeed Holler and Kendrick were one of few prior studies that also included older adults), whereas most work on videoed conversations used younger adults. It is therefore possible that differences in behaviour were due to participant age. Since older adults, and those with hearing loss that commonly occurs with age, particularly benefit from visual information in addition to audio during speech listening (Winneke and Phillips, 2011; Puschmann et al., 2019), our older participants may have prioritised turning early to better follow the conversational flow. However, as the behaviour of younger adults alone has not been explored in this real conversation paradigm, such age differences are highly speculative.

Interestingly, while prior work using videoed conversation demonstrated that more degraded audio led to more delayed orientation to the upcoming talker (Hidalgo et al., 2022), we did not see an effect of noise level on mean head turn timing. This may again be due to enhanced cues that talkers use to signal that they are ending/starting when talking in a real conversation in noise. Since the work by Hidalgo and colleagues used speech that had been recorded in an ideal environment but was subsequently manipulated to sound degraded to an observer, they would not have captured any compensatory behaviours that interlocutors naturally use to support conversation success (and potentially turn-end/turn-start prediction). Indeed in conversation, interlocutors have been shown to adjust both their speech level, known as the Lombard effect (Garnier et al., 2010; Zollinger and Brumm, 2011), and their gesture, a potential multimodal variation of the Lombard effect (Trujillo et al., 2021), to improve communication in noisy conditions. There is thus a large range of multimodal strategies that people can use to support communication in noise, and strategy use varies according to the acoustic environment (Hadley et al., 2019, 2021). Such adjustments are not possible when audio is recorded in one environment and then subsequently manipulated—hence our participants may have used vocal and gestural adjustments to make turn following easier, while participants in Hidalgo et al. did not do so due to being recorded in quiet.

However, while we did not see a slowing of head turns in noise, we did see an increase of variability; head movements had a 10% larger standard deviation at 78 dB than at 60 dB, meaning they were more spread around speech onsets/offsets when the noise level was higher. This distinction between the mean and the standard deviation of orientation timing could reflect that while predictions were made in a similar manner regardless of noise, the temporal precision of these predictions may have been reduced for degraded speech. Interestingly, this complements recent work demonstrating not only slower but also more variable timing of spoken turns in noise (i.e. focusing on the upcoming talker’s use of prediction rather than the observer), as well as more variable timing of spoken turns for people with hearing loss (who themselves experience degraded speech input, Sørensen et al., 2019).

**Figure 8:**
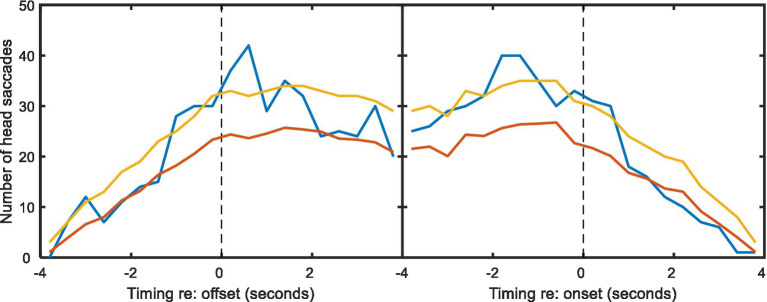
As Figure 5, but for the data from Lu et al. (2021).

A rather similar experiment to that of Hadley et al. (2021) has been reported by Lu et al. (2021). Although they used the same spatial configuration and also included a background noise manipulation, their paradigm differed in that head movement was only recorded from one interlocutor, as the other two were confederates. We have conducted a similar analysis on those data (presented in Figure 8 below). From a series of 20-min conversations in different noise levels we saw qualitatively similar patterns in the data: in non-overlapping speech, the majority of head turns occurred in the gap between talkers, and there were a number of seemingly predictive turns. However, Figure 8 shows that these data display weaker trends, likely since their study (1) produced considerably less HM data (<10 h of data from individuals, rather than 29 h) and (2) used lapel microphones requiring voice activity to be manually labelled (posing particular problems in noisy conditions). When comparing the number of HMs consistent with conversation following with the number required for statistical significance (Figure 8, yellow lines), it is clear that while the Lu et al. data are consistent with those from Hadley et al., they do not provide independent statistical support.

While analysis of the Hadley et al. data demonstrates predictive use of head turns in conversation, we cannot determine the signals that listeners used to identify that a turn was about to switch. Predictive turns could have been driven by a number of different cues, since during conversation the behaviour of each interlocutor feeds into the behaviour of every other interlocutor. For example, when one talker is coming to the end of their turn, they may convey signals that their turn is ending, leading a responder to prepare their retort. This would be a case of a prior talker signalling their turn end. An alternative is that while a turn is ongoing, a listening participant thinks of a point they want to insert, and that the listener’s signal that they want to jump in leads the current talker to yield the floor. Thus, in both cases, a turn ended (potentially signalled by turn-end cues), but in the latter case, this was a result of ‘floor request’ signals from a listener. Such a communication feedback loop (Beechey et al., 2020) makes it challenging to tie head turns to particular turn-end/turn-start cues. Nonetheless, the fact that there were up to 7% more anticipatory turns for overlapping speech (i.e. interruptions) than non-overlapping speech suggests that the cues from the upcoming talker may be particularly salient. In addition, we did not use a linguistic analysis to tie orientation switch timing to specific types of turns. While prior work demonstrates that predictive turns occur more often for questions than other sorts of exchanges (Casillas and Frank, 2017), it is possible that features of other exchanges such as directly addressing a specific listener or referencing to a topic raised by somebody else would also increase predictive turns. Future studies with more constrained behaviour manipulation may elucidate such issues.

In conclusion, this paper re-analysed an existing dataset of older adults engaged in triadic conversation to provide evidence that listeners can anticipate when a speaker will finish a conversational turn. This work demonstrates the value of head movements in assessing interlocutors’ focus of attention around a conversational turn switch, and indicates the propensity of listeners to orient towards an upcoming talker before they start talking. Such findings could be highly relevant for hearing technology. Hearing devices use beamformers to amplify specific regions around the wearer’s head while dampening others, and currently have a wide beam to amplify most of what is in front of the user. Recent work, however, has begun to explore the potential benefits of narrower beamformers (Soede et al., 1993; Best et al., 2015; Hládek et al., 2019), increasing the importance of understanding head movements around turn switches (to avoid dampening critical information). Greater understanding of natural behaviour in conversation would therefore provide a foundation for such technological advances, while also providing insight into the cognitive processes that are involved in the complex task of holding a conversation.

## Data availability statement

Publicly available datasets were analysed in this study. This data can be found at: osf.io/w6t3y.

## Ethics statement

Ethics approval was obtained from the West of Scotland Research Ethics Committee (09/S0704/12). The patients/participants provided their written informed consent to participate in this study.

## Author contributions

LH and JC contributed to the conception and design of the paper. JC performed the statistical analysis, wrote sections of the manuscript, contributed to the manuscript revision, and read and approved the submitted version. LH wrote the first draft of the manuscript. All authors contributed to the article and approved the submitted version.

## Funding

LH is supported by a UKRI Future Leaders Fellowship [grant number MR/T041471/1]; and the Medical Research Council [grant number MR/X003620/1]. JC is supported by the Engineering and Physical Sciences Research Council [grant number EP/S030298/1]. Funders did not play a role in study design, data collection and analysis, decision to publish, or preparation of the manuscript. The University of Nottingham provided open access publication fees.

## Conflict of interest

The authors declare that the research was conducted in the absence of any commercial or financial relationships that could be construed as a potential conflict of interest.

## Publisher’s note

All claims expressed in this article are solely those of the authors and do not necessarily represent those of their affiliated organizations, or those of the publisher, the editors and the reviewers. Any product that may be evaluated in this article, or claim that may be made by its manufacturer, is not guaranteed or endorsed by the publisher.
